# Concealed penis: A review of multilevel classification and surgical reconstruction techniques

**DOI:** 10.1002/bco2.470

**Published:** 2025-01-02

**Authors:** Bo‐yu Xiang, Jing‐xuan Peng, Xue‐jun Shang, Xiong‐bing Zu, Dong‐jie Li

**Affiliations:** ^1^ Department of Urology Xiangya Hospital, Central South University Changsha China; ^2^ Department of Andrology, Jinling Hospital, School of Medicine Nanjing University Nanjing Jiangsu China; ^3^ National Clinical Research Center for Geriatric Disorders Xiangya Hospital, Central South University Changsha China

**Keywords:** classification, concealed penis, surgical reconstruction

## Abstract

Concealed penis (CP), also known as hidden or buried penis, is an external genital deformity in which a normal‐sized penis is covered by skin, subcutaneous tissue or fat tissue in the prepubic area, leading to abnormal exposure. This condition has significant morbidity and a negative effect on certain aspects of the patient's quality of life, including but not limited to hygiene, micturition, self‐image and sexual function. Current classification systems for CP are heterogeneous, but most of these further classify CP based on their division into congenital concealed penis (CCP) and adult‐acquired buried penis (AABP). The aetiology and pathogenesis of this disease are understood to some extent, but the specific underlying mechanisms need further research. Although conservative treatment may be effective for some children with CCP, surgical intervention is still the main treatment for other children with CCP and AABP. There is no ‘gold standard’ surgical treatment for CP, but there are various surgical methods available; therefore, individualized surgical plans should be created after a comprehensive evaluation based on the classification and aetiology of CP patients.

## 1 INTRODUCTION

Concealed penis (CP), alternatively referred to as hidden penis or buried penis, is classified as an external genital malformation wherein the normal‐sized phallus is obscured by the presence of skin, subcutaneous tissue and/or adipose tissue in the prepubic region, which causes abnormal exposure of the phallus.^1^ CP, trapped penis and webbed penis are considered inconspicuous penis, which refers to a group of anatomical abnormalities in which the penis looks smaller than expected.^2^

CP is most common in children, and the prevalence of CP in China is 0.68%.^3^ Matsuo et al.^4^ reported that the prevalence of CP in Japanese newborns was 2%–5%, decreasing to 0.3% by the age of 4–5 years. Although relatively rare, CP can also occur in adults and is called adult‐acquired buried penis (AABP). The most common cause is obesity, and the other causes include severe genital lymphoedema, genital sweatshirt and postcircumcision scar tissue hyperplasia.^5^, ^6^ Hyguchi et al.^7^ reported that the obesity rates in adult males undergoing surgical treatment for CP can reach 87%, and as the obesity rates increase among adolescents, the prevalence of AABP is likely to increase as well.^8^ Prolonged concealment of the penis within the skin can cause a series of physical and mental issues, such as abnormal penile development, urination difficulties, recurrent infections, sexual dysfunction, depression and low self‐esteem; therefore, timely and effective intervention is needed.^9^, ^10^, ^11^ Although scientists have performed many studies on CP in recent years, there is no consensus on the classification, aetiology or treatment of CP, which has led to certain problems in clinical diagnosis and treatment.

In this review, we propose to introduce the classification of CP, divide CP into congenital concealed penis (CCP) and AABP, and systematically describe the aetiology and pathogenesis of the two types of CP and the progress on diagnosis and surgical methods, with the aim to provide references and guidance for urologists and paediatric surgeons.

## 2 CLASSIFICATION

There are many classification methods for CP, but there is controversy about which is the ideal method. However, it can be clearly stated that the appropriate subsequent treatment methods are closely related to the different subtypes. Crawford et al.^12^ conducted their first systematic classification of buried penis based on clinical observations in 1977, using the degree of penile exposure as a distinguishing criterion. They classified buried penis into two types: fully buried penis and partially buried penis. Maizels et al.^13^ considered buried penis and CP as different manifestations of the same disease and developed a detailed classification system for inconspicuous phallus from a pathological perspective. In addition to buried penis, this system also includes webbed penis, trapped penis, micropenis and diminutive penis. In 1999, Casale et al.^14^ divided CP into three types based on pathology: Type 1, CCP; Type 2, CP due to scarring from a previous surgery; and Type 3, complex cases involving excessive obesity (Table 1). Several scientists^15^ have classified CP into the following three subtypes from the aspects of concealment degree and morphology. Mild: the glans penis and some parts of the corpus cavernosum can be palpated; Moderate: the glans penis can be palpated, but the corpus cavernosum of the penis cannot be; Severe: the corpus cavernosum or glans penis can be palpated only when fingers press on both sides of the bottom of the penis. From a morphological perspective,^16^ CP can be divided into (1) patients with longer inner prepuce (LIP) only; (2) patients with LIP combined with an abnormal distal attachment of the penile ligament; and (3) patients who had, in addition to the above, excess suprapubic fat.

**TABLE 1:** Classifications of CCP.

Grade	Crawford et al.	Maizels et al.	Casale et al.	Hadidi et al.
1	Concealed penis	CP	CCP	Abnormal LIP
2	Partially CCP	Webbed penis	Concealed penis due to previous surgery	Abnormal LIP and abnormal distal attachment of the penile ligaments
3	Completely CCP	Trapped penis	Complex cases involving excessive obesity	Abnormal LIP, distal ligament attachment, and excess suprapubic fat
4	Partial penoscrotal webbing	Micropenis		
5	Complete penoscrotal webbing			

Abbreviations: CCP, congenital concealed penis; CP, concealed penis; LIP, longer inner prepuce.

To standardize and better categorize patients with AABP (Table 2), Tausch et al.^16^ initially proposed a classification system. Grade 1 patients have viable penile skin and required limited surgical intervention. Grade 2 patients do not have available penile skin and require a certain degree of skin grafting or reconstruction. Grade 3 patients have genital lymphoedema necessitating significant resection of diseased tissue and reconstructive procedures. This particular grading system does not account for the extent of abdominal obesity.^16^ Mirastschijski^17^ proposed another classification system based on the location of the glans and penile shaft in relation to the adjacent tissue. In Type 1, the shaft is covered by excess tissue; in Type 2, the shaft is invaginated but can be manually exposed; in Type 3, the shaft is completely invaginated; and manual exposure of the glans/shaft is not possible. Pariser et al.^18^ presented a classification system based on their single‐centre experience, and the types were graded according to the complexity of the surgical repair used for correction. In Type 1 patients, the penis needs to be removed with a local flap; Type 2 patients need a penile skin graft; Type 3 patients need scrotoplasty or scrotal reduction; Type 4 patients need escutcheonectomy for excessive suprapubic fat pad; and Type 5 patients need complete abdominal panniculectomy. Hesse and his colleagues^19^ published the Wisconsin AABP classification system, which is the first study to attempt to classify the disease process itself and provided the most common surgical steps associated with the type of AABP to validate the system's clinical utility. Kevin et al.^20^ proposed an improved AABP classification system based on the above studies, that is, the penis‐abdominal‐scrotum classification system, which divides AABP into 13 types according to seven major anatomical markers and governs follow‐up diagnosis and treatment procedures according to this classification scheme.

**TABLE 2:** Classifications of AABP.

Grade	Tausch et al.	Mirastschijski et al.	Pariser et al.	Hesse et al.
1	Need limited surgical intervention	The shaft is covered by excess tissue	Need to remove the penis with a local flap	AABP due to skin deficiency, iatrogenic scarring, and/or diseased penile skin
2	Need skin grafting or reconstruction	The shaft is invaginated but can be manual exposed.	Need a penile skin graft	Excess abdominal skin and fat
3	Need resection of the diseased tissue and reconstructive procedures	The shaft is complete invaginated, and manual exposure of the glans/shaft is not possible.	Need scrotoplasty or scrotal reduction	Excess skin and fat with diseased penile skin
4			Need escutcheonectomy	Grade 3 plus severe scrotal oedema
5			Need complete abdominal panniculectomy	

Abbreviation: AABP, adult‐acquired buried penis.

In summary, the classification of CP can provide theoretical guidance for the selection of treatment methods, and its purpose is to develop an individualized surgical plan. However, there is no certain classification method that can be used as a unified standard in clinical practice. We recommend to classify CP into CCP and AABP to conduct in‐depth research and develop personalized surgical methods.

## 3 AETIOLOGY AND PATHOGENESIS

**FIGURE 1:**
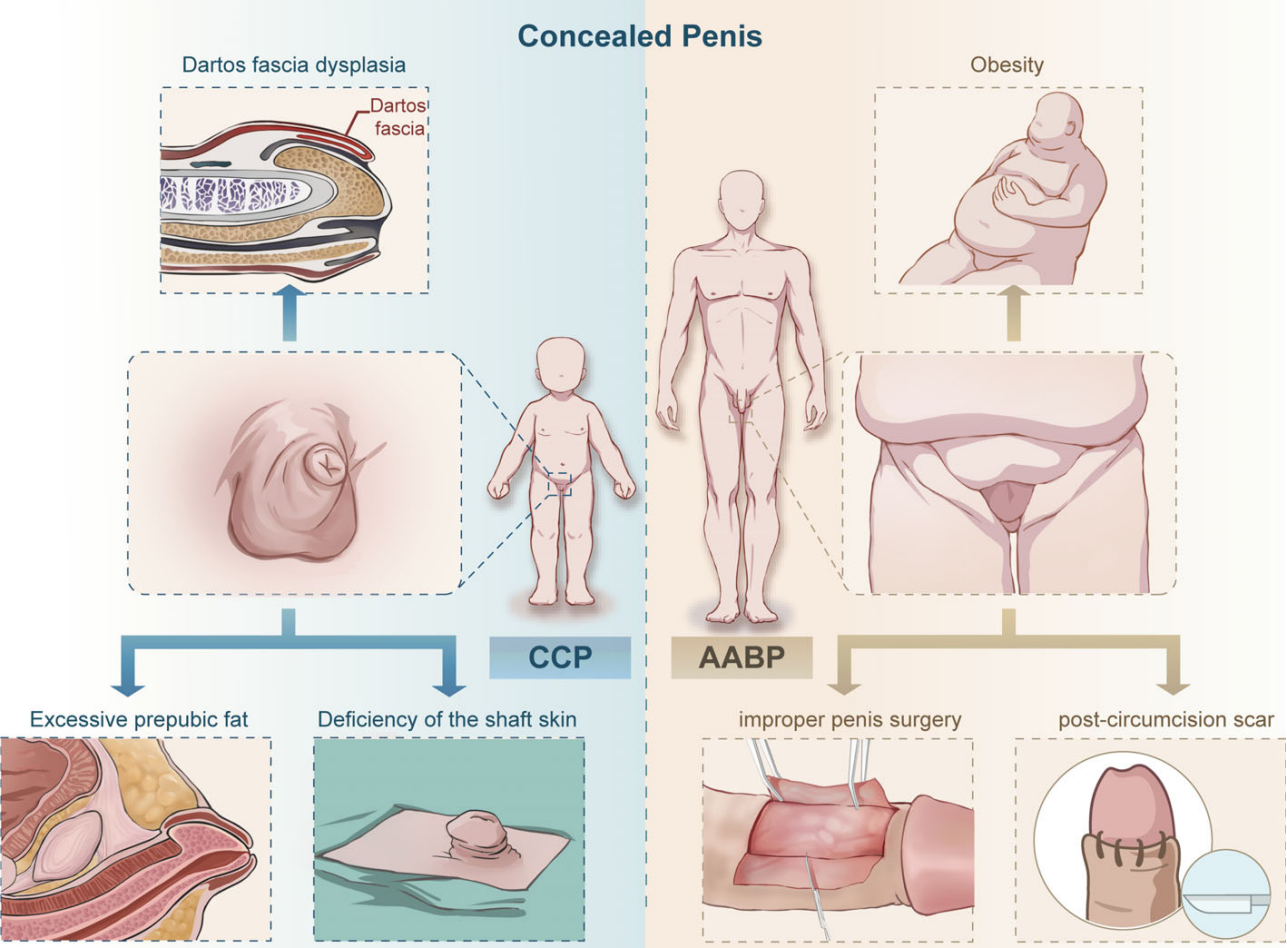
Schematic overview of the aetiology of CP. CP, concealed penis.

The aetiology of CP is not clear, and different scholars have different opinions. The generally recognized aetiology of CP is depicted in Figure 1 and will be further elucidated in the following discussion separately.

The aetiology of CCP is not fully understood, and different causes have been suggested, as follows: (1) abnormal elastic fibres restrict penile extension^1^, ^14^, ^21^, ^22^; (2) penile fascia dysplasia or fat accumulation between the dartos fascia and deep fascia^23^; (3) absence of the root ligament^24^; and (4) deficiency of the shaft skin.^25^ According to Spinoit et al.,^26^ the majority of CCP cases are caused by a lack of elasticity of the dartos fascia. Several studies have shown that up to 74% of patients with CCP have histologic abnormalities of the dartos fascia, which is consistent with Spinoit's description. Cromie et al.^1^ suggested that the dartos fascia is underdeveloped and attaches only to the corpus cavernosum of the penis and that retraction of the penile skin results in the inability of the skin to adequately attach to the body of the penis. Hadidi et al.^27^ suggested that abnormalities in the attachment points of the suspensory ligaments of the penis can also cause CCP. The most widely accepted hypothesis is that CCP is caused primarily by abnormally thick and fibrous bands of dartos fascia.^26^ These bands, which are more rigid and dense than normal, extend from Scarpa's fascia, attaching themselves to either the middle or distal dorsal shaft of the penis and exerting a tethering force on the cavernosal bodies proximally. Additionally, the fat pad between the dartos fascia and deep fascia cannot be attached to the penis shaft from the root of the penis but can be directly attached to the anterior part, even the tip of the penis shaft. This creates a triangular shape between the penile dartos fascia, the penile shaft and the pubic symphysis, resulting in the conical appearance of the penis that is characteristic of CCP. This is the primary cause of a CP in infants and toddlers, while in adolescents, CP is mainly caused by an excessive accumulation of subcutaneous fat in the perineal area.^21^

However, AABP is considered to be involved in a different process from that of CP seen in the paediatric patients.^28^ Although some dysgenetic dartos tissue may play a role in paediatric patients, it cannot be regarded as the main factor in adults. Obesity is the leading cause of AABP.^29^ With subcutaneous adipose tissue accumulation, the penis, which is anchored to the pubis by suspensory ligaments, appears to lose its subjective length. This accumulation of fat can also cause the mons to enlarge, overshadowing the penis.^5^ Consequently, a moist environment forms under the overlying tissue, fostering fungal and bacterial growth. This condition triggers chronic inflammation and skin deterioration, eventually leading to scarring and contracture.^30^, ^31^ In prolonged cases, such as when there is persistent inflammation, the lesions may even progress to squamous cell carcinoma of the penis.^32^ In addition to obesity, various comorbidities and conditions, such as diabetes mellitus (DM), lichen sclerosis and genital lymphoedema, can aggravate this syndrome.^33^ Each of these factors can either cause local tissue damage or impair proper wound healing.

## 4 DIAGNOSIS

The diagnosis of CP relies mainly on clinical manifestations and physical examination.

### 4.1 Clinical manifestations

CCP patients often complain of a short penis. Patients with preputial stenosis may have dysuria, urine retention, urinary tract infection^21^, ^34^ and so forth. As children grow older, they may have secondary psychological problems such as inferiority, autism and anxiety.^34^ For AABP patients, providers should not only pay more attention to any comorbidities, including smoking status, nutritional status, adjacent skin conditions, Diabetes Mellitus, chronic lymphoedema and autoimmune disorders,^10^, ^35^ that may affect postoperative healing but also should carefully query patients about their urologic history and surgical history to fully evaluate their condition.^36^, ^37^

### 4.2 Physical examination

The currently accepted physical examination criteria^38^ for CP include the following: (1) the penis is short or buried; (2) the foreskin is ‘beak‐like’ or ‘hill‐like’; (3) the penis skin is relatively insufficient and unevenly distributed; and (4) the normal penis body is exposed when the skin of the penis root is pushed backward by the hand and the penis body quickly retracts after release. (5) The angle of the penis and scrotum is obtuse. (6) The testicles are well‐developed; and (7) there is excessive prepubic adipose tissue. Pestana^5^ and Harrison^8^ suggested that patients be physically examined in the standing and supine positions, allowing the provider to determine the condition of the abdomen, suprapubic region and genital area. Recently, Kobiljon et al. introduced a concealed index for CCP children. The authors measured the stretched penile length (SPL), penile circumference (PC) and penile length above the baseline skin level (BPL) using a ruler (cm) and the testicular volume using an orchidometer (mL). The concealed index according to the SPL (CI) was defined as the BPL/SPL, and the concealed index according to circumference (CIc) was defined as the BPL/PC; these values might be useful and objective parameters for checking the severity of CP and evaluating the postoperative outcome of CP repair.^39^

### 4.3 Diagnostic examination

B‐ultrasound can measure the length of the penis and the size of the testes, and the levels of testosterone, oestrogen, chorionic gonadotropin, luteinizing hormone, gonadotropin and other hormones need to be tested in some children to confirm the diagnosis. Some scholars suggest that preoperative B‐ultrasound should be performed in the inguinal region to determine whether there is an inguinal hernia.^40^

### 4.4 Differential diagnosis

In clinical practice, CCP is often overlooked or misdiagnosed. Its differential diagnosis mainly includes micropenis, penile dysplasia, phimosis and epispadias. Micropenis and penile dysplasia are caused by endocrine defects or chromosome abnormalities, resulting in a clinically small penile shaft with less erectile strength. Phimosis is characterized by a narrow preputial opening that does not expose the glans penis, which is contraindicated for CCP surgery. Typical signs of congenital malformation can be observed and distinguished from CCP.

## 5 TREATMENT

There are still many controversies about the treatment of CCP. There is no unified standard on whether to choose conservative treatment or surgical treatment or how to choose the treatment plan and timing. However, for adult patients, once diagnosed, they should be treated with surgery as soon as possible.^37^ The appropriate surgical methods should be selected according to the aetiology and severity of the disease in different patients.

### 5.1 Conservative treatment (only for CCP)

The principle of conservative treatment for CCP is to choose a personalized treatment plan according to the aetiology of the disease, and the decision on which treatment should also be chosen after considering the clinical manifestations and the wishes of the guardians or children with good judgement ability. Metcalfe et al.^41^ reported that in some children with CCP, the amount of testosterone that is released increases during adolescence, the dartos fascia is gradually rearranged under the influence of hormones, the prepubic fat also decreases or is redistributed, and the penis will naturally elongate. Thus, it is recommended to watch and wait for most patients and consider surgical treatment only for those patients who cannot self‐heal. Srinivasan et al.^42^ confirmed this view and reported that many prepubertal children with CCP secondary to excessive prepubic or suprapubic fat do not require surgical correction. The excess fat pads in most children become thinner or even disappear as they age, and the penis is exposed after the patient reaches puberty. Therefore, if there is no recurrent balanoposthitis,^24^ urinary tract infection, hygiene problems, erectile pain, voiding difficulties, including urine spraying and urine dripping^13^ and psychological problems (such as excessive anxiety) in preschool children with CCP, they can be treated with watch and wait and conservative treatment, regardless of whether the glans can be exposed or not, and can be evaluated when they are at school age.^43^

For children with CCP and obesity, initial conservative treatment can be considered, which focuses on weight loss (potentially in collaboration with endocrinologists and nutritionists to develop a weight loss plan) and maintaining local hygiene (guidance on cleaning the upturned foreskin). As the penis develops and the amount of prepubic fat decreases, successful weight loss in most children can lead to significant improvement in penile appearance during adolescence.^41^ Some scholars suggest conservative treatment for children with CP after penile surgery. Palmer et al.^44^ used betamethasone to treat 16 children with CP due to cicatricial contracture postcircumcision. After treatment, 11 patients showed softening of the scars, two required simple scar resection, and only three needed surgical correction. However, in general, surgery is still the main means of treatment because of the uncertain effect of conservative treatment.^45^

## 6 SURGICAL CORRECTION

### 6.1 Surgery timing

Regarding infants with CCP, LI Z et al.^46^ believe that surgical correction at the infancy stage is not suitable because, regardless of which surgical method is chosen, the surgical risk is substantial, the surgical complexity is challenging, and the surgical outcome is constrained. Some scholars^41^ have proposed that the most appropriate surgical timing is at age 12 to 14 because at this age, the disease progresses through the early stage of child sexual development, during which the androgen concentration continues to increase. Under the stimulation of testosterone, the rapid development of the penis will cause great changes in its appearance (whether in diameter or length). Children with CCP may heal on their own at this age, but if they do not, timely surgical intervention is needed. According to Herndon et al.,^47^ children need surgical intervention once diagnosed. Long‐term burial of the penis will cause varying degrees of physical and mental damage to children. As children enter puberty, penile erection will occur relatively frequently. During the erection process, the penis will be pulled by the abnormal development of the carnosus membrane, resulting in erectile pain. In severe cases, it may cause penile curvature deformities.^48^

Based on the literature review, guidelines and consensus, the authors believe that the timing of surgery for children with CCP should be considered according to the wishes of patients and parents and the operability of surgery. Doctors should pay close attention to the progress of the disease, and health education about CP should be provided to children and their guardians to improve their understanding of the disease and overcome adverse psychological emotions. For the vast majority of these children, the appropriate timing of surgery is before and after school age, and appropriate timing cannot only reduce the adverse physiological and psychological effects of CP but also facilitate surgical operation and postoperative recovery. For mild, moderate or obese children, waiting and watching are recommended, and a re‐evaluation should be performed after the penis has developed to a certain extent or after weight loss. For those patients who recover after puberty, no special treatment is needed, and for children those who cannot recover after puberty, surgery can be selected according to their general health. For patients with secondary recurrent balanoposthitis or urinary tract infection, surgical intervention should be performed in a timely manner, regardless of age. However, due to the lack of long‐term follow‐up data and high‐level clinical evidence, the optimal timing of surgery for CCP remains to be further studied. Once patients are diagnosed with AABP, they should undergo surgery as soon as possible.^37^

### 6.2 Indications and contraindications

At present, the accepted surgical indications are as follows: (1) severe stenosis of the external prepuce; (2) severe penile skin deficiency; (3) dysuria and balanoposthitis; and (4) effects to the appearance and mental health of children.^49^, ^50^, ^51^, ^52^

The contraindications for surgery include (1) a CP combined with hypospadias, epispadias or disorders of sex development, (surgery alone should not be performed before the treatment of comorbidities); (2) the onset of penoscrotal infection or systemic infectious disease; and (3) coagulopathy, severe bleeding tendency that has not been effectively controlled, or systemic diseases making the patient not able to tolerate surgery.

### 6.3 Surgical procedures

**FIGURE 2:**
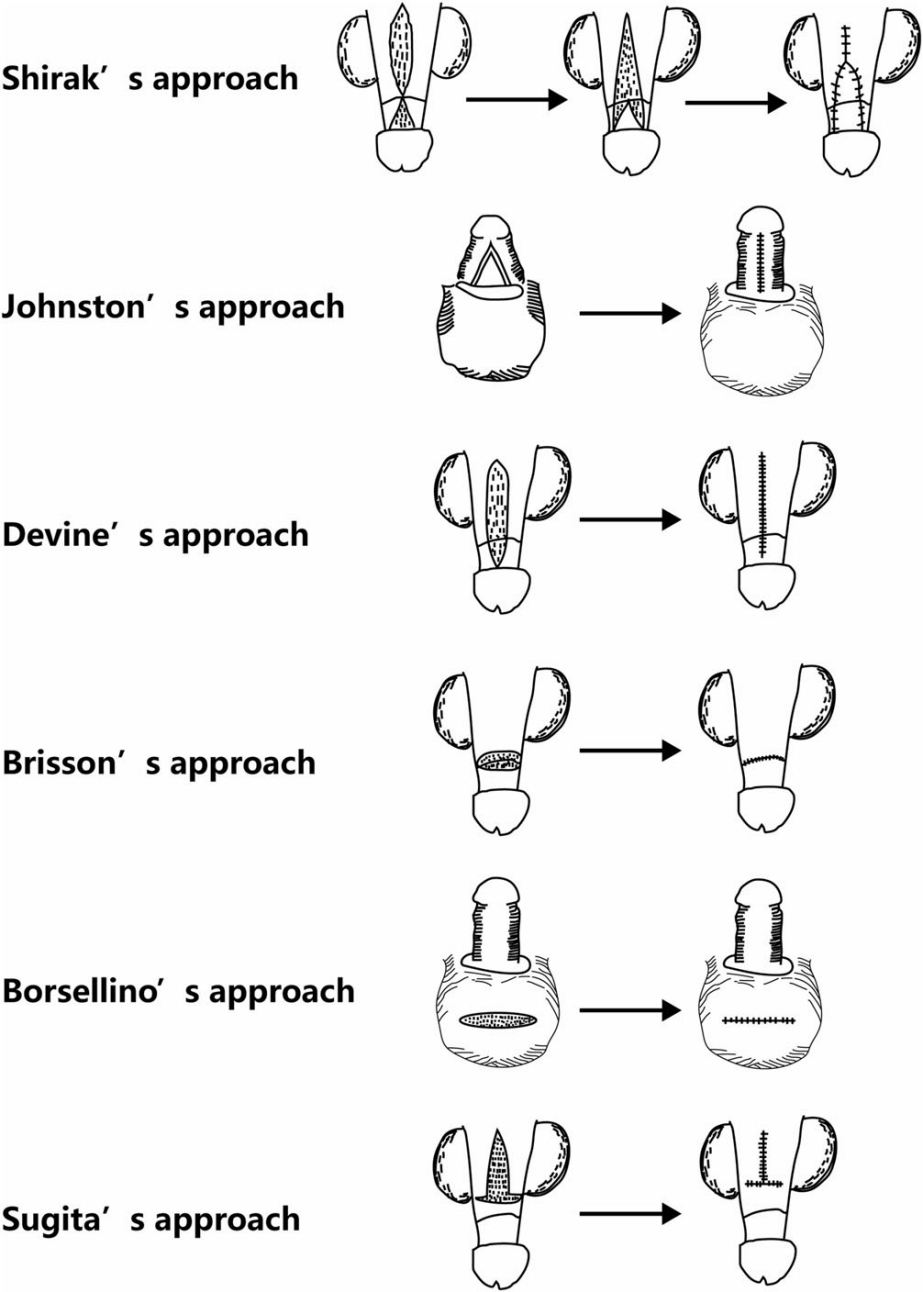
Simple schematic diagram of surgical approach of CP. CP, concealed penis.

There is no ‘gold standard’ surgical treatment for CP, but there are various surgical methods available (Table 3 and Figure 2). Common surgical methods include the Shiraki procedure that was first described in 1975, the Johnston procedure and the Wollin procedure, first described in 1990, the Devine procedure, first described in 1992, the Brisson procedure, first described in 2001 and the Sugita procedure, first described in 2009. In recent years, modified surgical procedures have been developed on the basis of the above procedures, and various auxiliary procedures, such as CP surgery combined with suprapubic liposuction, have also emerged. Although there is marked variability in the surgical procedures, a systematic approach to surgery should be applied in each patient. The steps are as follows: (1) repair of phimosis, including complete exteriorization of the shaft and removal of abnormal adhesions; (2) liposuction and lipotomy of the pubic region (if necessary); (3) scrotoplasty and reconstruction of the penopubic and penoscrotal angles^53^; and (4) coverage of the shaft. The specific techniques are described as follows:

**TABLE 3:** The classic surgical approach for treating CP.

	Key points	Merit	Demerit
Shirak's approach	V‐incisions at the 0, 4, 8 o'clock positions on the penile shaft and prepuce were connected in a Y‐shape, extending proximally to cover the skin defect.	Fully preserved penile skin	Abnormal membrane left, penis partly retracted, poor foreskin appearance, large cut risks flap ischemia from overseparation
Johnston's approach	A circular incision is made at the base of the penis, extending to the tunica albuginea, to address the treatment of the suprapubic fat pad, with the skin adhered to the pubic periosteum.	The incision is small and obscure but effectively prevents penile retraction.	Risk of severe postsurgery swelling due to venous and lymphatic blockage; potential damage to the dorsal penile vessels and nerves; untreated fibrous cords result in poor penile straightening and appearance.
Devine's approach	The prepuce is cut longitudinally on the dorsal side of the penis and circumcised on both sides of the narrow ring. The penile skin is degloved completely to the root of the penis, the fibrotic carnosus tissue is removed, and the penile skin is fixed to the tunica albuginea.	The dorsal blood vessels of the penis are well protected	The incision is small, cannot be completely exteriorized; the penoscrotal angle is not moulded.
Brisson's approach	A circular incision around the coronal sulcus is used to fully expose the penis to its base. Dysplastic tissue or fat is removed, excess inner plate is trimmed, and the skin at the base is secured to the tunica albuginea and deep fascia.	The abnormal fibrous cord is fully removed; the penis is extended sufficiently, a low retraction rate.	The coverage of the ventral skin defect affects the postoperative appearance.
Borsellino's approach	A circular incision is made at the narrow ring and is made deeper into the scrotum to fully exposed the penis to remove the abnormal fibrous cord. If needed, the suspensory ligament is cut, and the penis is reattached to reconstruct the phallopubic and phalloscrotal angles.	The abnormal fibrous cord is fully removed, good aesthetic degree.	Possibility of vascular and nerve damage; not suitable for severe CP patients
Sugita's approach	The narrow ring of prepuce is cut ventrally, and an inverted ‘T’ incision is made on the dorsal side. After adequate degloving and fixation, the dorsal prepuce is transferred from both sides to the ventral side.	High flap survival rate; full use of the penile skin is made; and the frenulum prepuce is kept intact.	Severe postoperative oedema and long recovery time

Abbreviation: CP, concealed penis.

Shiraki et al.^54^ introduced a procedure for CCP treatment, drawing inspiration from the Y‐V procedure used in hypospadias repair in children. This technique involves making longitudinal incisions at the 0, 4 and 8 o'clock positions on the penis and a V‐shaped incision on the inner prepuce, connecting these incisions in a Y‐shaped to extend to the proximal part of the penis, thus addressing the skin defect. This method focuses on reshaping the penile skin without removing the underlying abnormal carnosus membrane. Postsurgery, some penile retraction occurs due to the surrounding fat, leading to suboptimal outcomes.^54^ Chen C et al.^55^ refined the Shiraki procedure by (1) employing cross sutures on the inner and outer penile skin flaps to widen the constricted preputial opening and (2) excising the abnormal carnosus membrane and suturing both sides of the penile base to prevent retraction. These modifications resulted in improved penopubic and penoscrotal angles in CCP children, significantly enhancing penile exposure and boosting parental satisfaction. Building on this, Bo Peng et al.^56^ implemented scrotal flap transplantation, which markedly increased the postoperative penile length compared with that obtained with the original Shiraki method. This approach yielded consistent skin coloration and natural‐looking penoscrotal and penopubic angles and was associated with a lower complication rate compared to that with the traditional Shiraki approach.

In 1990, Johnston introduced a novel surgical approach for CCP children who had CCP secondary to excessive prepubic fat, and the approach involved a circular incision at the penis base, meticulous separation of the tunica albuginea, attachment of the entire penile subcutaneous tissue layer to the tunica albuginea, glans exposure, and fat removal from the pubic area.^49^ Termed the Johnston procedure, it involved an annular fixation that was shown to offer enhanced reliability. However, the risk of this method is that it can compromise the majority of superficial veins and lymphatic drainage systems on the penis, potentially harming the dorsal nerves and blood vessels. Such damage can lead to persistent oedema, potentially impacting adolescent erectile function and adult sexual health.^57^ Additionally, the failure to address abnormal fibrous tissue with this procedure results in minimal improvements in penile straightness and leaves much to be desired in terms of aesthetic outcomes.

The Devine procedure is a well‐established surgical technique, and this technique involves making a longitudinal incision on the dorsal side of the penis to preserve the dorsal neurovascular bundle.^58^ The procedure involves (1) making longitudinal incisions on the inner and outer foreskin panels along the dorsal midline, transverselyizing the original longitudinal incision, then extending it, and circumcising the remaining skin; (2) excising underdeveloped cord‐like carnosus tissue; and (3) attaching the penile skin to the tunica albuginea. Its limitations include the need for a small incision, leading to a limited operative field; difficulty in separating and removing fibrous tissue; and the risk of incomplete penile exteriorization. The modified Devine procedure involves a longitudinal incision on the ventral side of the penile shaft, and an incision is made that extends from the constricted preputial ring to where the narrowing ends. A transverse incision is made near the coronal sulcus on the dorsal side, after which the incision is angled toward each side, connecting ventrally to the initial incision.^59^ This method mirrors the original steps of Devine's exteriorization and fixation, with the addition of removing excess skin from the ventral inner prepuce in an inverted V shape and suturing the remaining inner plate longitudinally. The foreskin's inner and outer plates are then sutured together to encase the penis. The benefits of this technique include cutting the constricting ring from the ventral side for complete foreskin release via oblique circumcision, which facilitates glans and penile shaft exposure, and this technique provides a broader surgical view and allows for thorough chorda removal. Ziyi Zhang et al.^60^ monitored 56 patients who had the postmodified Devine procedure, and significant penile lengthening, absence of retraction at 12 weeks post‐operation, and heightened parental satisfaction were noted in that study.

Wollin^34^ introduced a novel surgical approach known as the ‘Wollin surgical technique’, utilizing a pedicled island flap. This technique involves (1) fully releasing and/or excising the abnormal fascia and dorsal fibrous cords to completely expose the corpus cavernosum and (2) separating a vascularized island flap from the inner preputial plate, creating an opening at the flap's mesentery base, and relocating it to the ventral side of the penis to address preputial defects. The advantage of this procedure lies in its ventral incision, which allows the surgeon to optimize the use of the penis's outer plate for dorsal and proximal coverage and allows the inner prepuce flap to be transferred to the ventral side, ensuring a circumcision‐like appearance post‐operation. Building on this, numerous researchers have globally integrated this technique with penis and penile root skin fixation (the Brisson procedure). In 2001, Brisson^61^ introduced a unique vertical mattress suture technique at the penis base for skin attachment involving suturing the prepubic fascia and the tunica albuginea at the 2, 10 and 12 o'clock positions, followed by penile root skin suturing at six specific points. Su Q^62^ modified this technique by employing multiple needles and directions for penile fixation, significantly reducing postoperative retraction and achieving satisfactory outcomes. The fixation technique evolved from six points to four points at 3, 6, 9 or 12 o'clock or two points at various positions,^62^, ^63^ effectively preventing urethral damage and minimizing postsurgical prepuce oedema.

The Borsellino^64^ technique involves the addition of a midscrotal longitudinal incision, facilitating direct penis extraction after shaft exteriorization. With this approach, ventral skin incisions are avoided, abnormal dartos fascia fibrous tissue is fully excised, and the penoscrotal and penopubic angles are reconstructed by fixing the penis dorsally and ventrally. This auxiliary incision has gained wide acceptance in various modified surgeries.

In 2009, Sugita et al.^50^ suggested a method involving a longitudinal incision on the dorsal foreskin's inner plate, which was rotated to the ventral side to cover skin deficits, and this technique mirrors Devine's fixation steps. The Sugita procedure, characterized by its simplicity in design and execution, is broadly applicable for most cases of penile skin insufficiency. Key aspects include transferring the inner prepuce flanks ventrally, ensuring a short flap transfer distance with a broad base and robust blood supply to decrease the risk of flap necrosis, and positioning the surgical scar ventrally for an aesthetically pleasing outcome and high patient satisfaction. Some modifications of this technique involve anchoring the dermis of the dorsal skin of the penis root to the tunica albuginea, enhancing the appearance of the penis and reducing the incidence of retraction.^49^

Skin grafting involves reconstructing the surface of the penis by transplanting skin from other body parts to the penis after removing scar tissue or excess skin. Flap grafting, akin to skin grafting, involves the removal of subcutaneous tissue beneath the skin, ensuring the blood supply of the grafted area. Skin grafts are frequently used for multiple penile skin deficiencies, with the glabrous skin of the lateral thigh or the suprapubic region being common donor sites. Donatucci,^65^ Han,^66^ and Warren et al.^67^ reported a preference for full‐thickness skin grafting to minimize scar formation. Strother et al.^37^ reported that for AABP patients with severe penile skin deficiency, medium‐thickness skin grafting led to a greater survival rate of the graft and greater postoperative satisfaction with the penis's appearance. However, flap transplantation is more suited for small skin defects, such as those on the ventral penis. Several researchers^8^ suggest treating severe CP by transferring flaps from the dorsal prepuce, utilizing the inner prepuce plate and dorsal penis skin to fill in any ventral gaps, which enhance the appearance satisfaction of the penis and reduces the chances of penile flexion deformities. One study^66^ successfully corrected AABP by advancing the musculocutaneous scrotal flap technique, yielding a pleasing postoperative appearance and high patient satisfaction for ventral skin defects not covered by dorsal prepuce flaps. For large dorsal skin defects, transferring the ventral or scrotal flap to the dorsal side has been an effective correction method.

Liposuction can increase the degree of penile exposure by suctioning excessive fat around the prepubic region, and this technique is suitable for patients with local adipose accumulation and sufficient skin elasticity.^68^ Pubic lipectomy removes the fat pad above the pubis to increase the length of the penis, and this technique is often performed in combination with other surgical procedures, to achieve aesthetic results.^69^ For CP patients with severe fat accumulation in the lower abdomen or pubic region, a better postoperative appearance can be obtained with liposuction or pubic lipectomy.^68^ During liposuction, the area is stained with methylene blue, swelling fluid is injected until the skin becomes white and hard, and a small incision is made with a sharp knife and then sucked in a fan shape until satisfactory. Suprapubic lipectomy with an Ω‐shaped incision reportedly removes the prepubic fat pad, reverses exteriorization, loosens the abnormal fibre cord, and fixes the root of the penis. The appearance of the lower abdomen is then natural and flat, and the surgical incision is concealed. No complications, such as penile retraction, occurred, and patient satisfaction was high.^69^

In recent years, with the deepening of research on the aetiology and pathological mechanism of CP, many scholars have continuously optimized the surgical procedures on the basis of the above classic surgical methods and proposed various modified surgical methods, which have achieved good clinical efficacy and patient satisfaction. The modified Devine and modified Brisson techniques have been favoured by an increasing number of urologists and paediatric surgeons because of their advantages, namely, simplicity of operation, excellent postoperative efficacy and low complication rate; additionally, these methods have gradually become mainstream surgical methods for the treatment of CP.^60^, ^70^, ^71^ In addition to the classic surgical methods and the corresponding modified surgical methods, there are many simple surgical procedures and techniques that are easy to perform. For example, Paolo Caione et al.^51^ reported the ‘two angles’ surgery in which the junction of the penis and scrotum skin are longitudinally cut on the ventral side, the penis is freed at the level of the penile root, and the dartos fascia is fixed at the junction of the penis and scrotum on both sides to the penopubic tissue to reconstruct the penoscrotal angle, firmly fix the extended penis, and reduce the incidence of penile retraction. The learning curve is shorter, and circumcision can be avoided. Zhang H et al.^72^ proposed the idea of the ‘anatomical’ repair; that is, the dartos fascia is divided into deep and shallow layers, and the superficial tissue is thick and loose. The deep tissue is thin and dense, less vascularized, more fixed, and closely connected to Buck's fascia. The penile scrotal angle can be reconstructed by circular resection of the deep fartos fascia, and the Buck fascia is sutured and fixed to the superficial dartos fascia and subcutaneous tissue at the junction of the penis and scrotum at the 5 o'clock and 7 o'clock positions, which can effectively reduce the rate of penile retraction. In general, individualized surgical plans should be created after performing a comprehensive evaluation based on the classification and aetiology of CP patients.

### 6.4 Postoperative complications and management

#### 6.4.1 Bleeding and hematoma

Bleeding and hematoma are frequent complications after surgery and typically result from inadequate haemostasis during surgery or unstable penile dressing postsurgery; these often manifest within 24 h postsurgery. Their management includes applying pressure bandages to alleviate the hematomas and, if necessary, inserting a drainage tube.^5^, ^6^

#### 6.4.2 Oedema

Research indicates that up to 60% of patients may experience oedema following CP, primarily due to lymphatic drainage dysfunction.^73^ This condition often improves over weeks or months.^34^, ^47^ A study reported that utilizing indocyanine green (ICG) for lymphatic assessment and during surgery can significantly lower the risk of developing postsurgical lymphoedema.^74^ In addition, excessive preservation of the preputial frenulum during surgery can lead to oedema. Similarly, overretention of the preputial inner plate or tissue from the transfer flap may result in a skirt‐like effect or excessive growth on the underside of the penile skin and subcutaneous tissue. If the above conditions are serious, secondary surgery is needed.^50^

#### 6.4.3 Flap necrosis

Flap necrosis can occur due to a minimal flap pedicle, inadequate flap blood supply or excessive subcutaneous tissue removal, leading to blood supply issues and localized necrosis of the flap or penile skin. Mild cases can be treated with oral antibiotics and regular dressing changes or debridement and suturing if needed, allowing natural healing. Severe cases require debridement and flap transplantation.^13^

#### 6.4.4 Incisional infection

Incisional infections are usually caused by excessive postoperative bleeding, delayed dressing changes or uncontrolled preoperative prepuce infection and present with redness, pain, pus, abscess, incision dehiscence and penile skin necrosis. Enhanced dressing management, ensuring drainage patency and anti‐infection treatments are crucial.^13^

#### 6.4.5 Ischemia and necrosis of the glans

When the dressing is too tight after surgery or when the blood vessels supplying the glans are injured during the operation, the patient's glans becomes dark, swollen and painful, and the patient has severe sensation loss or complete necrosis.^75^ Immediate removal of any tight bandages when it occur and careful avoidance of the glans blood vessels during surgery are necessary.

#### 6.4.6 Scar formation

Severe surgical‐induced damage to penile skin and soft tissue can inhibit natural repair, leading to fibrous tissue replacement and scar formation, potentially causing penile curvature deformities. In some cases, severe scarring at the penoscrotal angle necessitates secondary surgical correction due to the resultant penile curvature.^46^

#### 6.4.7 Penile retraction

This may be linked to obesity or improper fixation of the skin dermis at the penis base and penile cavernous body root, resulting in the penis retracting into prepubic subcutaneous tissue, which is sometimes more severe than it was before surgery. Reoperation to secure the base of the penis is often required.^56^

#### 6.4.8 Painful erection

Adolescents may experience painful erections postsurgery due to improper fixation of the penis root, and its treatment focuses on symptomatic and supportive care. If pain persists, reoperation for penis fixation may be considered.^53^

#### 6.4.9 Fistula

Intraoperative damage to the ventral urethra can cause urethral fistula, a serious complication necessitating secondary urethral repair surgery. CP with membranous urethra cases is rare but requires preoperative identification to avoid the development of a postoperative fistula from improper surgical techniques. The repair methods depend on the location and size of the fistula.^53^

### 6.5 Follow‐up

For children with CCP, long‐term follow‐up is crucial. Parents and children should be informed that a CP is merely a developmental issue of external genitalia exposure, which often normalizes after treatment, thereby reducing psychological stress.^52^ Follow‐ups focus on satisfaction levels regarding penile appearance, complemented by objective measures such as developmental scales and monitoring for complications such as abnormal urination, penile retraction and local oedema. In addition, attention should be paid to the secondary sexual characteristics of children in adolescence, sexual life and fertility after marriage. Conservative treatment follow‐ups are advised at age 5, before puberty (~11 years) and during adolescence. Surgical patients should be seen at 3, 6 and 12 months post‐operation, annually for 2 years, and at least 3 years if symptoms persist. The follow‐up period includes questions about surgical satisfaction, psychological changes and sexual function. Surgical satisfaction is a key aspect for evaluating outcomes such as size, shape and urination. Greater parental satisfaction is obtained with the treatment of younger patients, and patient satisfaction is significantly increased after surgery and correlates with improved penile aesthetics.^47^ Dissatisfaction arises from suboptimal surgical results, aesthetics or issues such as penile retraction.^55^ Psychological changes are assessed by using the Paediatric Penile Perception Scale (PPPS) and Child Behaviour Checklist, as well as anxiety and depression scales. Awareness of potential prepuberty psychological shifts is vital, necessitating early intervention.^19^ Postsurgical psychological impacts, potentially extending into adolescence, warrant attention. Notably, some patients reported symptoms of postsurgical organ rejection, underscoring the need for prolonged psychological follow‐up.^53^ Studies^76^ have shown that surgery may affect the sexual function of patients to a certain extent. However, due to the absence of extensive, long‐term studies, the impact of CP surgery on sexual function has not been determined.

Repairing AABP is a nuanced and diverse process aimed at enhancing quality of life and genitourinary functionality. Increasing evidence indicates that patients opting for surgery often experience substantial life improvements. Follow‐up surveys revealed that 85% to 92% of patients would opt for the surgery again, and 74% to 83% of patients report a beneficial impact on their lives.^77^ Functionally, a notable enhancement in performing daily activities is generally achieved.^78^ Specifically, in terms of genitourinary function, positive outcomes have been reported. Rybak et al.^9^ observed a marked improvement in voiding capabilities and a significant reduction in depression scores. Hughes et al.^79^ documented notable enhancements in sexual function post‐ABP correction. These improvements in urinary and sexual functions are further supported by a recent case series from Pittsburgh by Theisen et al.^80^ Collectively, the current limited data suggest that patients who were carefully selected to receive AABP correction tend to report positive and lasting results from the AABP correction.

## 7 CONCLUSION

Despite the advancements in various areas, significant scientific challenges remain in understanding the classification, causes, mechanisms and treatment options for CP, necessitating extensive future research. Key areas include as follows:

1. Classification of CPs, particularly CCP and AABP: As research has progressed, the CP classification has diversified, considering not only appearance, morphology and pathology but also the relationship with the surrounding tissues and surgical complexity. Further distinctions between CCP and AABP have emerged. The absence of a universally accepted classification complicates diagnosis and treatment. The field could greatly benefit from future research in which a CCP‐ and AABP‐based classification system is developed using high‐quality clinical evidence to optimize treatment selection.
2. Understanding the aetiology and pathogenesis of CP: While some causal mechanisms are known, the intricacies of these processes remain unclear. The genesis of congenital CP, in addition to acquired factors, is particularly elusive. Investigating the specific reasons for abnormal penile skin and tissue development through embryology and molecular studies could elucidate these mechanisms.
3. Standardizing CP treatment: Surgery is the primary treatment, but with the various methods available, each with its own pros and cons, there is no standardized approach. This stems from a lack of comprehensive clinical study evidence and long‐term follow‐up data. Future efforts to establish a standardized treatment protocol through high‐quality research, tailoring to individual patient needs, would be invaluable.

In conclusion, treating CP goes beyond mere surgical intervention; it involves reconstructive efforts that significantly impact patients' physical and psychological well‐being and their future. Therefore, clinicians must develop personalized treatment plans based on a thorough understanding of the treatment principles, aiming to enhance patient and family satisfaction.

## AUTHOR CONTRIBUTIONS

Bo‐yu Xiang and Jing‐xuan Peng were a major contributor in writing the manuscript. Xiong‐bing Zu made a substantial contribution to discussion of content. Dong‐jie Li and Xue‐jun Shang reviewed and edited the article before submission. All authors read and approved the final manuscript.

## CONFLICT OF INTEREST STATEMENT

The authors declare that they have no competing interests.
